# The Role of Circulating Extracellular DNA in Patients with Bladder Cancer: Clinical Associations and Exploratory Links with Heart Rate Variability

**DOI:** 10.3390/cancers18182952

**Published:** 2026-09-12

**Authors:** Patrik Palacka, Hana Kováčová Ilijew, Iveta Mikolášková, Magda Suchánková, Mária Paulovičová, Beáta Rondziková, Boris Kollárik, Peter Celec, Ľuba Hunáková

**Affiliations:** 1 2nd Department of Oncology, Faculty of Medicine, Comenius University and National Cancer Institute, Klenová 1, 833 10 Bratislava, Slovakia; 2 Cancer Research Institute, Biomedical Research Center of the Slovak Academy of Sciences, Dúbravská Cesta 9, 845 05 Bratislava, Slovakia; 3 Institute of Immunology, Faculty of Medicine, Comenius University, Odborárske Námestie 14, 811 08 Bratislava, Slovakia; 4 2nd Department of Urology, Faculty of Medicine and Bory Hospital, Comenius University, Ivana Kadlečíka 2, 841 03 Bratislava, Slovakia; 5 Institute of Molecular Biomedicine, Faculty of Medicine, Comenius University, Sasinkova 4, 811 08 Bratislava, Slovakia; 6 Institute of Pathophysiology, Faculty of Medicine, Comenius University, Sasinkova 4, 811 08 Bratislava, Slovakia

**Keywords:** bladder cancer, extracellular DNA, nuclear DNA, mitochondrial DNA, nuclease

## Abstract

Background: Extracellular DNA (extracellular DNA) is a promising biomarker for tumor burden and systemic inflammation. However, its clinical significance in urothelial carcinoma remains unclear. This study evaluated plasma extracellular DNA (nuclear and mitochondrial fractions) and DNase activity in patients with bladder cancer (BC) in comparison to healthy controls, and explored their associations with tumor stage, survival, and heart rate variability (HRV) as a non-invasive marker of autonomic regulation. Methods: We prospectively analyzed 84 subjects: 63 patients with urothelial carcinoma (52 non-muscle-invasive [NMIBC]; 11 muscle-invasive [MIBC]) and 21 healthy controls. Plasma extracellular DNA was quantified fluorometrically, while ncDNA and mtDNA fractions were assessed via quantitative real-time PCR. DNase activity was determined using the single radial enzyme diffusion assay. Clinical associations were evaluated through group comparisons, multivariable logistic regression, survival analysis, and correlation with HRV parameters. Results: Patients with BC exhibited slightly but significantly higher extracellular DNA compared to healthy controls (by 9%); ncDNA showed a similar trend. Regarding disease progression, ncDNA demonstrated a stronger association with tumor stage than total extracellular DNA, with the highest concentrations observed in patients with MIBC. In multivariable logistic regression models adjusted for age, BMI, sex, smoking, and alcohol consumption, extracellular DNA remained independently associated with BC status (OR 5.99, 95% CI 1.43–25.12, *p* = 0.015). Log-transformed ncDNA was also associated with BC status, although the model was constrained by missing data. No significant differences in overall survival were observed when stratified by cutoff values of extracellular DNA, ncDNA, mtDNA, or DNase activity. Notably, while extracellular DNA correlated significantly with several HRV parameters in healthy individuals (after FDR correction), this physiological coupling was absent in patients with BC. Conclusions: Circulating extracellular DNA and ncDNA are independently associated with BC, with ncDNA showing a stronger correlation with tumor stage. While prognostic value regarding survival was not confirmed in this cohort, the absence of extracellular DNA–HRV correlations in patients, despite significant associations in healthy controls, suggests the loss of physiological coupling between circulating DNA and autonomic regulation. Further longitudinal studies are needed to validate the clinical utility and biological interpretation of these markers.

## Simple Summary

This study investigated plasma extracellular DNA—including nuclear (ncDNA) and mitochondrial (mtDNA) fractions—and DNase activity in 63 bladder cancer (BC) patients and 21 healthy controls. The findings reveal that circulating extracellular DNA and ncDNA are independently associated with BC, with ncDNA showing particularly strong associations with advanced tumor stages (muscle-invasive BC). While survival outcomes did not significantly differ based on these markers, a notable physiological coupling between extracellular DNA and heart rate variability observed in healthy controls was absent in BC patients. These results suggest that circulating DNA and autonomic regulation become uncoupled during malignancy, highlighting extracellular DNA and ncDNA as promising biomarkers for tumor burden and systemic changes, though further longitudinal studies are required to confirm their full clinical utility.

## 1. Introduction

Bladder cancer (BC) is one of the most common malignancies worldwide and remains a clinically heterogeneous disease associated with significant morbidity and mortality. According to recent global estimates, BC accounts for more than 600,000 new cases and more than 220,000 deaths annually [1]. Urothelial carcinoma is the predominant histological subtype, comprising approximately 90% of all BCs [2]. Clinically, the disease is commonly classified as either non-muscle-invasive BC (NMIBC), encompassing Ta and T1 tumors, or muscle-invasive BC (MIBC), which carries a higher risk of progression, systemic dissemination, and cancer-related mortality. Despite advances in cystoscopic surveillance, diagnostic imaging, and histopathological evaluation, there remains a critical need for minimally invasive biomarkers to enhance early detection, improve biological risk stratification, and facilitate the monitoring of tumor-associated systemic alterations.

Extracellular DNA (extracellular DNA), often referred to as circulating cell-free DNA in plasma-based research, has emerged as a promising biomarker in oncology. It is released into circulation through various mechanisms, including apoptosis, necrosis, active secretion, the formation of neutrophil extracellular traps, and shedding from proliferating tumor cells [3,4]. In patients with cancer, extracellular DNA reflects both tumor-derived and host-derived processes, encompassing tumor burden, tissue injury, inflammation, and immune activation [5,6]. Given that circulating DNA can be analyzed via blood samples, it has garnered significant interest as a key component of liquid biopsy strategies for diagnosis, disease monitoring, and the assessment of treatment response [7,8,9,10]. Importantly, extracellular DNA is not a single biologically uniform entity: its nuclear and mitochondrial fractions provide complementary clinical information. While nuclear DNA (ncDNA) originating from both, tumor and non-tumor cells, may more directly reflect tumor-cell turnover, tumor-associated inflammation or tissue damage, mitochondrial DNA (mtDNA) can in addition act as a damage-associated molecular pattern (DAMP) with pro-inflammatory properties.

In various malignancies, elevated concentrations of circulating DNA have been associated with advanced disease, tumor aggressiveness, and adverse clinical outcomes [11,12,13,14]. However, the clinical relevance of extracellular DNA and its subcellular origin in urothelial carcinoma of the bladder remains incompletely characterized. Beyond the mechanisms of DNA release, circulating DNA is significantly influenced by extracellular DNA clearance. Deoxyribonucleases (DNases), particularly DNase I and its related nucleases, degrade extracellular DNA and thus may modulate its biological and clinical impact. Experimental and translational evidence suggests that impaired clearance of extracellular DNA contributes to systemic inflammation, thrombosis, immune dysregulation, tumor progression, and metastatic spread. Conversely, DNase-mediated degradation can attenuate many of these processes. Therefore, the combined assessment of total extracellular DNA, ncDNA, mtDNA, and DNase activity may provide a more comprehensive, integrated view of extracellular DNA dynamics in BC than the measurement of total extracellular DNA alone.

Beyond tumor-derived biomarkers, cancer is increasingly recognized as a systemic condition characterized by alterations in immune, metabolic, and autonomic nervous system regulation. Heart rate variability (HRV) serves as a non-invasive proxy for autonomic function—specifically vagal modulation—and reduced HRV has been consistently reported in oncology patients, often correlating with disease burden, symptom severity, and poor prognosis [15,16,17]. Physiologically, the autonomic nervous system and systemic inflammatory pathways communicate bidirectionally via neuroimmune pathways, most notably the cholinergic anti-inflammatory pathway [18,19,20]. Under normal homeostatic conditions, vagal efferent activity monitors and attenuates systemic tissue damage and inflammatory cascades. Given that circulating extracellular DNA represents a real-time indicator of host-derived tissue injury, necrosis, and inflammatory activation, while HRV reflects the vagal control of systemic homeostasis, a functional coordination (coupling) between these two markers is expected in healthy states. In patients with malignancy, however, tumor-related stress and chronic systemic inflammation may disrupt or override this autonomic–inflammatory axis. Examining whether the correlation between circulating extracellular DNA and HRV indices is preserved or lost in BC can therefore provide valuable clinical insight into the integrity of host autonomic–inflammatory regulation during oncogenesis.

Therefore, the primary objective of this study was to comprehensively evaluate circulating extracellular DNA, ncDNA, mtDNA, and DNase activity in patients with urothelial carcinoma of the bladder in comparison to healthy controls. We aimed to determine whether these markers differ according to BC status and tumor stage, to assess their independent association with the presence of BC after adjustment for relevant clinical and lifestyle covariates, and to explore their relationship with overall survival (OS). In addition, we investigated the associations between circulating extracellular DNA and selected HRV parameters to explore whether the relationship between circulating DNA burden and autonomic regulation differs between healthy individuals and patients with BC.

## 2. Patients and Methods

### 2.1. Baseline Characteristics

Patients and healthy controls were enrolled between November 2021 and December 2023, with a data cut-off of 16 April 2026. At a median follow-up of 34.7 months (interquartile range [IQR]: 31.5–41.3 months), 14.3% of the patients had died.

Baseline demographic and clinical characteristics of the study cohort are summarized in Table 1. A total of 84 subjects were included, comprising 21 healthy controls, 52 patients with NMIBC, and 11 patients with MIBC. The groups were comparable in terms of age and sex distribution, although significant differences were noted in smoking status, which were subsequently included as covariates in the multivariable regression analyses.

### 2.2. Extracellular Circulating DNA and DNase Activity Analyses

At baseline, 6 mL of peripheral blood was atraumatically collected from the antecubital vein of each patient into EDTA tubes (BD Vacutainer^®^) during the morning hours immediately prior to the intervention. Within 2 h of venipuncture, the samples were initially centrifuged at 2000× *g* for 10 min at 4 °C. The upper plasma layer was carefully transferred to a fresh tube, avoiding the buffy coat, and subjected to a second centrifugation at 16,000× *g* for 10 min at 4 °C to remove residual cellular debris. The resulting cell-free plasma was aliquoted into 1-mL volumes and stored at −80 °C until further analysis.

Extracellular DNA and DNase activity were assessed as described before with slight modifications [21,22,23]. Samples were centrifuged at 1600× *g* for 10 min at 4 °C. Plasma was subsequently centrifuged at 16,000× *g* for 10 min at 4 °C to remove residual cells, cell debris, and apoptotic bodies. Extracellular DNA was isolated from 200 µL of the supernatant using the QIAamp DNA Mini Kit (Qiagen, Hilden, Germany) and eluted in 50 µL of ultrapure water. Total extracellular DNA concentrations were determined fluorometrically using the Qubit dsDNA High-Sensitivity Assay and a Qubit fluorometer (Thermo Fisher Scientific, Waltham, MA, USA). NcDNA and mtDNA were quantified using real-time PCR and the SsoAdvanced Universal SYBR Green Supermix (Bio-Rad, Hercules, CA, USA). The human β-globin gene was amplified as a marker of ncDNA (forward: 5′-GCT TCT GAC ACA ACT GTG TTC-3′; reverse: 5′-CAC CAA CTT CAT CCA CGT TCA-3′), whereas the mitochondrial D-loop was amplified as a marker of mtDNA (forward: 5′-CAT AAA AAC CCA ATC CAC ATC A-3′; reverse: 5′-GAG GGG TGG CTT TGG AGT-3′). Concentrations were calculated as genome equivalents (GE) per millilitre of plasma. DNase activity was measured using a modified single-radial enzyme-diffusion assay. Three microlitres of plasma were applied to a 1% agarose gel containing 2 mM MgCl_2_, 2 mM CaCl_2_, 20 mM Tris–HCl (pH 7.5), DNA substrate, and a fluorescent nucleic-acid stain. Following incubation for 16–20 h at 37 °C in the dark, the hydrolysis zones were visualized, their diameters measured using ImageJ 1.54e, and DNase activity calculated against a calibration curve prepared from serial dilutions of DNase I with known activity. Results were expressed in Kunitz units per millilitre.

Nuclear DNA (ncDNA) and mitochondrial DNA (mtDNA) concentrations were quantified using quantitative real-time PCR and expressed as genome equivalents (GE) per milliliter of plasma. For ncDNA, the single-copy human β-globin gene was amplified, where 1 ng of double-stranded DNA corresponds to approximately 303 genome equivalents (calculated based on an average haploid human genome mass of ~3.3 pg). For mtDNA, the mitochondrial D-loop region was amplified. Absolute copy numbers of the target regions were determined using standard curves generated from serial dilutions of standards with known concentrations. The final concentrations were subsequently adjusted for the initial plasma input volume (200 µL) and the elution volume (50 µL), and are expressed as genome equivalents per milliliter (GE/mL) of plasma.

### 2.3. Heart Rate Variability Assessment

Heart rate variability (HRV) was assessed as a non-invasive marker of autonomic regulation from electrocardiographic (ECG) recordings acquired at a sampling frequency of 1000 Hz using a Bittium Faros device (Bittium Corporation, Oulu, Finland). Recordings were obtained under standardized resting conditions following a 5 min stabilization. Participants were instructed to remain in a supine position in a quiet environment, avoid unnecessary movement and verbal communication, and breathe spontaneously.

The raw inter-beat interval (IBI) signal was visually inspected and preprocessed to remove artifacts, ectopic beats, and non-physiological intervals prior to HRV parameter calculation. HRV analysis was performed using Kubios HRV Premium software version 3.5.0 (Kubios Oy, Kuopio, Finland). Both time-domain and frequency-domain HRV indices were derived from the cleaned recordings. HRV parameters were selected a priori based on their established physiological relevance and widespread use in autonomic assessment. RMSSD and log-transformed high-frequency (HF) power were included as indices primarily reflecting parasympathetic (vagal) activity, while DCmod and ACmod were utilized to capture opposing aspects of heart rate dynamics, thereby providing complementary information on autonomic modulation. Log-transformed low-frequency (LF) power was included as a mixed index reflecting both sympathetic and parasympathetic influences. This focused selection allowed for a physiologically meaningful yet concise characterization of autonomic function while minimizing the risk of errors associated with multiple comparisons. Because LF and HF exhibited right-skewed distributions, they were log-transformed prior to statistical analysis.

Associations between circulating log-transformed extracellular DNA and selected HRV parameters were evaluated separately in healthy controls and patients with BC. For this exploratory analysis, log-transformed extracellular DNA concentrations were correlated with DCmod, ACmod, RMSSD, and log-transformed LF and HF power. The objective of this analysis was to determine whether the physiological association between circulating DNA burden and autonomic regulation differs between healthy individuals and patients with BC.

### 2.4. Statistical Analysis

Data distribution was assessed using the Shapiro–Wilk test. Normally distributed variables were compared using Student’s *t* test or analysis of variance (ANOVA), followed by Bonferroni or Tamhane post-hoc testing, as appropriate. Non-Gaussian variables were analyzed using the Mann–Whitney U test or the Kruskal–Wallis test with Dunn’s post-hoc correction. Fisher’s exact test was used to evaluate differences in proportions for categorical variables. Receiver operating characteristic (ROC) analysis was employed to determine the optimal extracellular DNA cut-off.

Correlations between circulating extracellular DNA and selected HRV parameters were performed using Spearman’s rank correlation coefficient. To account for multiple testing in the HRV correlation analyses, a false discovery rate (FDR) correction was applied using the Benjamini–Hochberg procedure. To address the right-skewed distribution of the circulating DNA markers (extracellular DNA, ncDNA, and mtDNA), we applied a logarithmic transformation. DNase activity, which exhibited an approximately symmetric distribution, was analyzed on its original scale. Although log-transformed values were utilized for exploratory and graphical representations, natural log-transformed variables were employed in the regression models. This distinction pertains strictly to data scaling and does not alter the directionality or statistical significance of the reported associations. The original units of the measured biomarkers are retained throughout the manuscript (extracellular DNA in ng/mL; ncDNA and mtDNA in GE/mL; DNase activity in KU/mL).

To formally evaluate whether the exploratory associations between circulating log-transformed extracellular DNA and selected HRV parameters differed significantly between healthy individuals and patients with BC, we performed a between-group comparison of the independent correlation coefficients. Spearman’s correlation coefficients (rho) from each group were converted to z-scores using Fisher’s r-to-z transformation. The standard error (SE) of the difference between the two independent correlation coefficients was calculated using the Spearman-specific correction factor of 1.06 to account for non-parametric distribution. The resulting z-test statistic was used to derive two-sided *p*-values. For comparative completeness, standard (unadjusted) Fisher’s z-tests were also conducted.

OS was defined as the time from transurethral resection of bladder tumor (TURBT) to the date of last follow-up or death from any cause. Survival probabilities were estimated using the Kaplan–Meier method and compared using the log-rank test. Patients were stratified according to either median biomarker values or optimal cutoff values derived from ROC analyses using the Youden index. All tests were two-sided, and *p* value < 0.05 was considered statistically significant. All statistical analyses were performed using NCSS, version 2024 (NCSS, LLC, Kaysville, UT, USA; 2024).

## 3. Results

### 3.1. Circulating DNA Concentrations in Bladder Cancer Patients and Their Association with Tumor Stage

**Figure 1:**
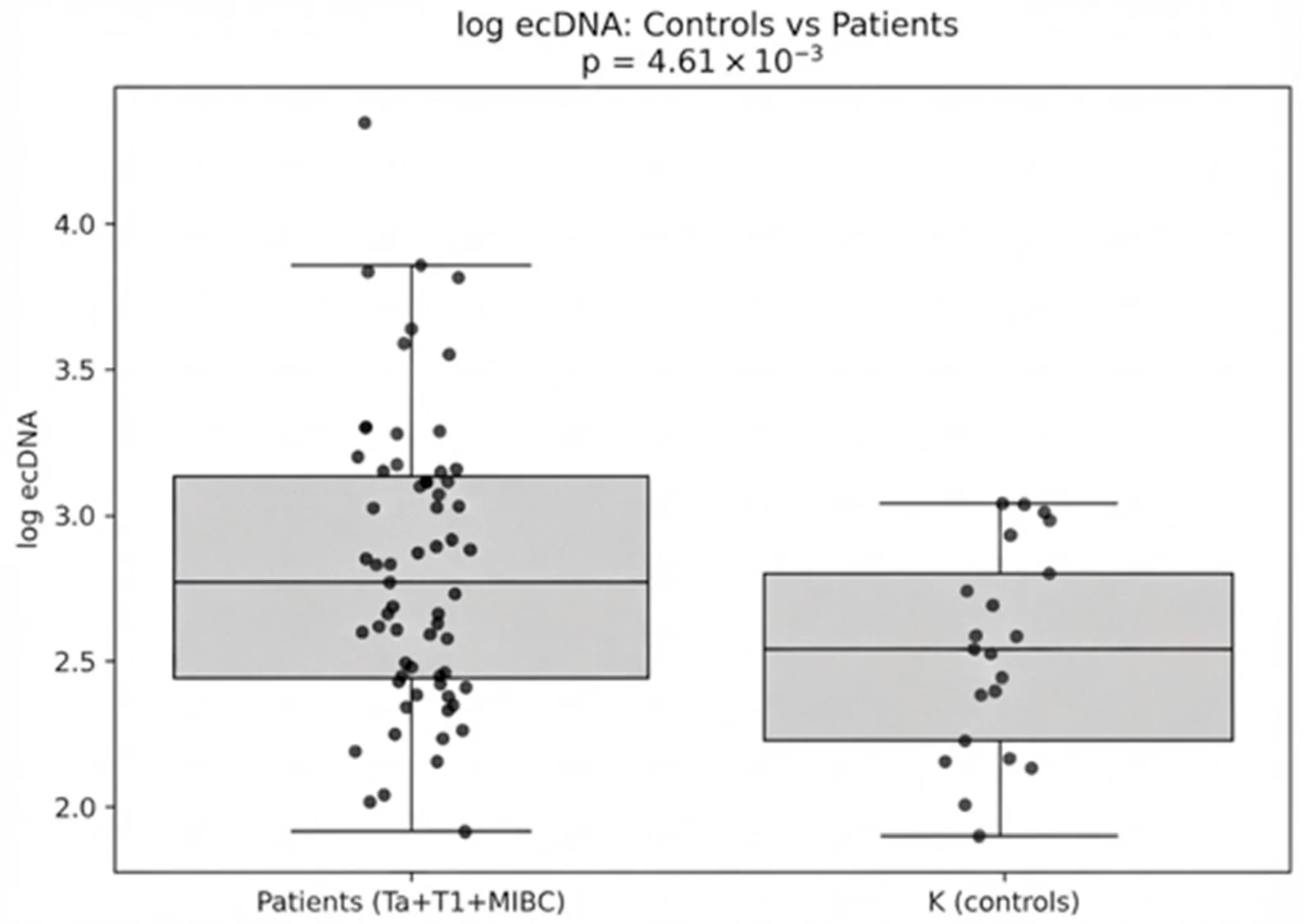
Log-transformed circulating extracellular DNA concentrations (extracellular DNA, ng/mL) in patients with bladder cancer and healthy controls.

Log-transformed circulating extracellular DNA (extracellular DNA) concentrations (ng/mL) differed significantly between healthy controls and patients with BC. Patients exhibited higher log-transformed extracellular DNA concentrations (median log value: 2.77 vs. 2.54, Mann–Whitney U test, *p* = 0.021), indicating an increased circulating DNA associated with malignancy (Figure 1).

**Figure 2:**
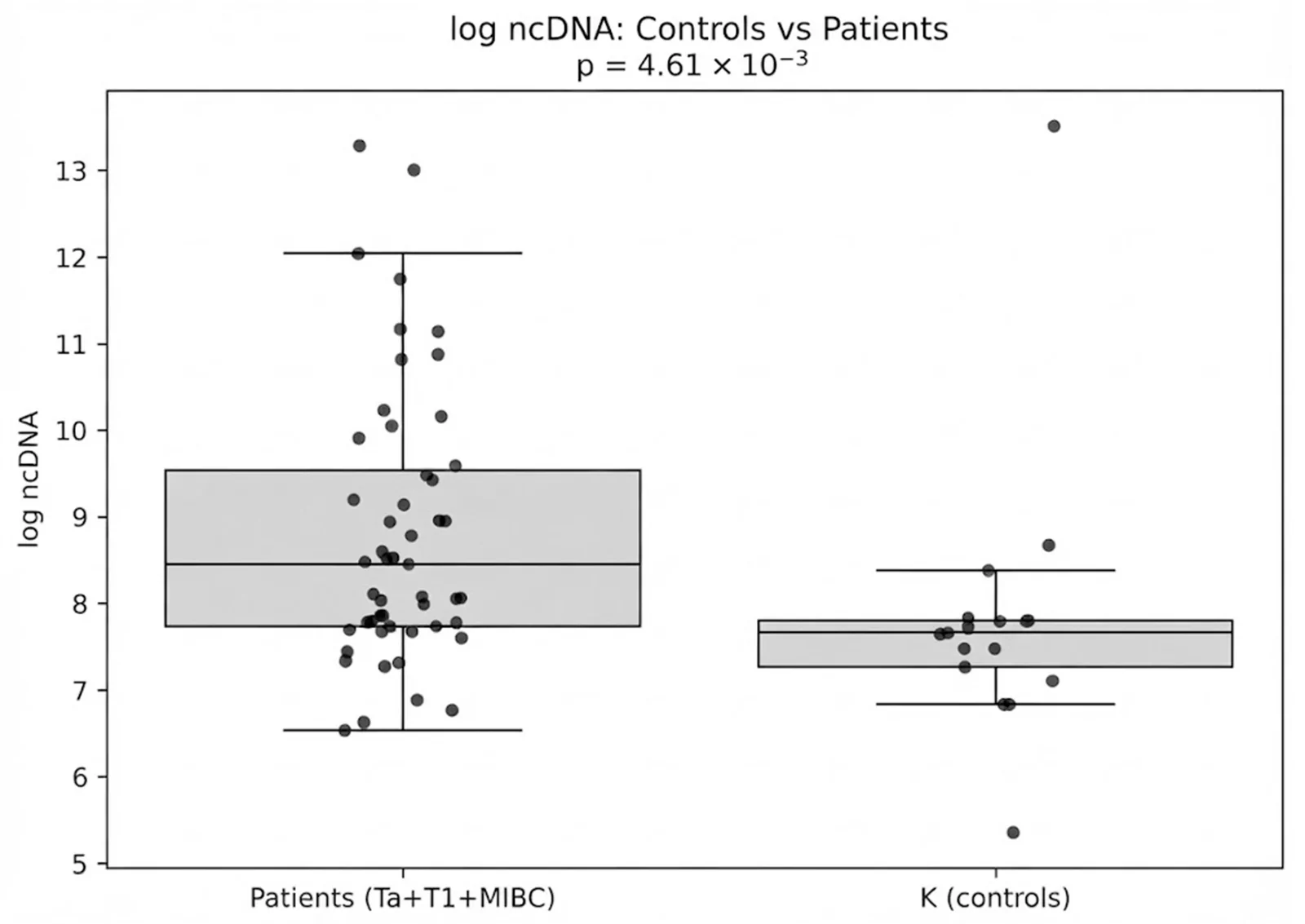
Comparison of log-transformed nuclear DNA concentrations (ncDNA, GE/mL) between healthy controls and patients with bladder cancer.

Similarly, log-transformed nuclear circulating DNA (ncDNA) concentrations (GE/mL) were elevated in patients compared to controls, further supporting the presence of an increased circulating DNA burden in the disease state. In contrast, no significant differences were observed between the groups for DNase activity (KU/mL) or log-transformed mitochondrial DNA (mtDNA) concentrations (GE/mL) (Figure 2).

**Figure 3:**
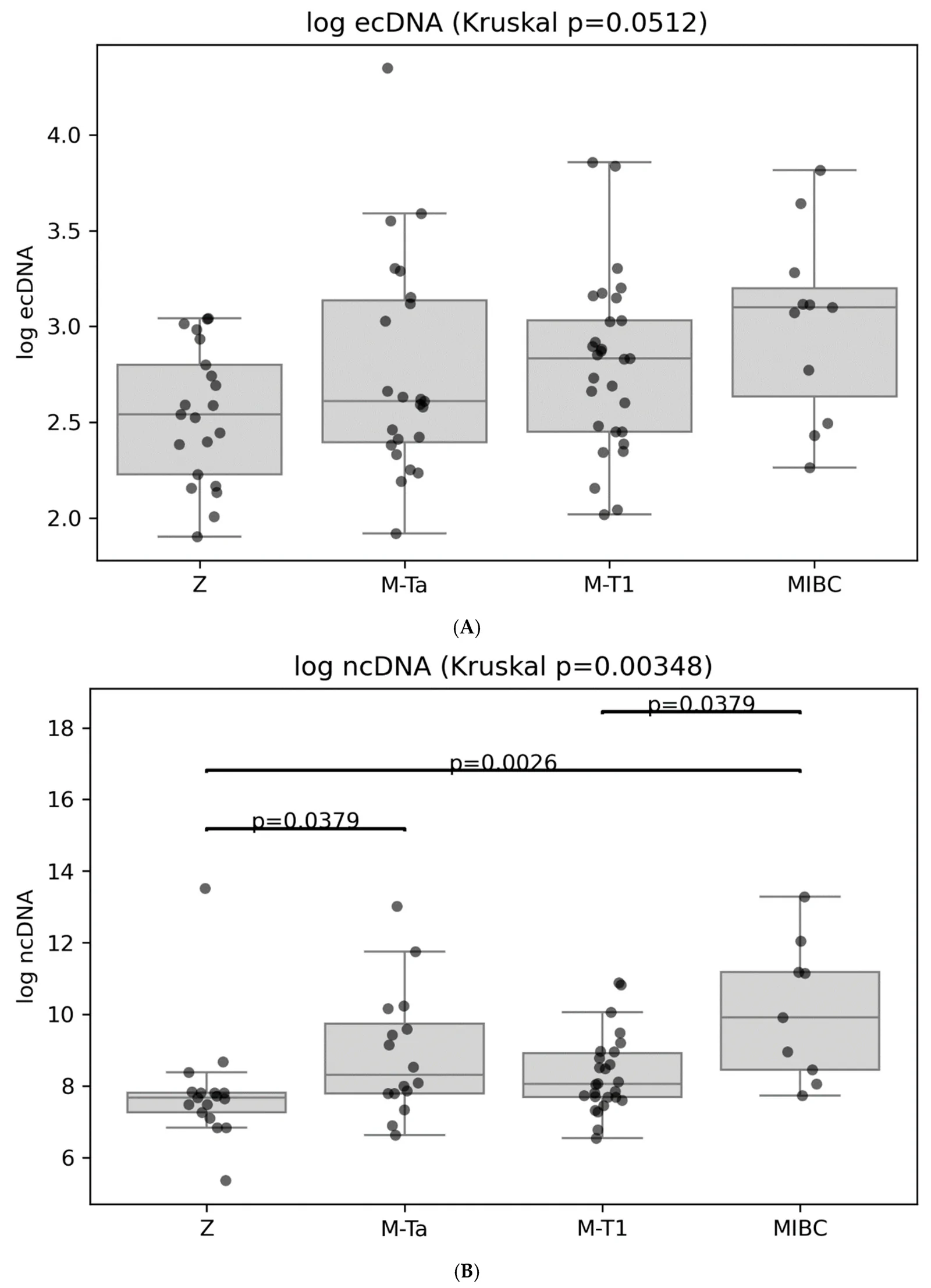
Circulating DNA markers across study groups. (**A**) Log-transformed extracellular DNA concentrations (extracellular DNA, ng/mL) across the four groups (controls, Ta, T1, and MIBC). (**B**) Log-transformed nuclear DNA concentrations (ncDNA, GE/mL) across the four groups.

Log-transformed extracellular DNA (extracellular DNA) concentrations (ng/mL) showed a trend toward variation across the four study groups (controls, Ta, T1, and MIBC), reaching borderline statistical significance in the Kruskal–Wallis analysis (Figure 3A).

Although log-transformed extracellular DNA concentrations appeared to increase with advancing disease stage, post hoc comparisons revealed limited statistically significant differences between individual groups.

In contrast, log-transformed nuclear DNA (ncDNA) concentrations (GE/mL) demonstrated a clear, statistically significant difference across the study groups. Post hoc Dunn’s analysis identified significantly higher log-transformed ncDNA concentrations in patients with advanced disease, particularly those in the MIBC group, compared with healthy controls and those with early-stage disease (Figure 3B).

Overall, ncDNA showed a more robust association with tumor stage than extracellular DNA, suggesting that ncDNA may serve as a more sensitive biomarker for disease progression in BC.

### 3.2. Logistic Regression Analysis

Univariable logistic regression analyses showed that higher levels of log-transformed extracellular DNA (OR = 3.88, 95% CI 1.17–12.81, *p* = 0.026), log-transformed ncDNA (OR = 1.44, 95% CI 1.01–2.06, *p* = 0.045), and daily alcohol consumption (OR = 2.87, 95% CI 1.04–7.90, *p* = 0.042) were associated with BC status. No significant associations were observed for age, BMI, sex, past smoking, or current smoking. To identify independent predictors of BC, multivariable logistic regression models were subsequently subjected to backward elimination. Variables with *p* > 0.10 were sequentially removed from the model according to the backward elimination procedure. For extracellular DNA, age, BMI, and sex were excluded during the selection procedure, while log-transformed extracellular DNA, smoking history, current smoking status, and daily alcohol consumption remained in the final model. Higher log extracellular DNA levels remained independently associated with BC (OR = 5.99, 95% CI 1.43–25.12, *p* = 0.015). The final model was statistically significant overall (Likelihood Ratio χ^2^ = 21.39, *p* = 0.0003) (Table 2).

Similarly, for ncDNA, age, BMI, and sex were excluded during backward selection, while smoking history, current smoking status, daily alcohol consumption, and log-transformed ncDNA were retained in the final model. Higher log ncDNA levels remained independently associated with BC (OR = 1.87, 95% CI 1.01–3.47, *p* = 0.048). The final model was statistically significant overall (Likelihood Ratio χ^2^ = 16.31, *p* = 0.0026) (Table 3).

### 3.3. Associations Between Circulating Extracellular DNA and Selected HRV Parameters

In the healthy group (*n* = 21), Spearman correlation analysis revealed significant associations between log-transformed circulating extracellular DNA concentrations and multiple HRV parameters (Table 4). Specifically, circulating extracellular DNA demonstrated moderate negative correlations with deceleration capacity (DCmod: rho = −0.51, *p* = 0.019, p_FDR = 0.041), the root mean square of successive differences (RMSSD: rho = −0.51, *p* = 0.019, p_FDR = 0.041), and log-transformed HF power (rho = −0.47, *p* = 0.033, p_FDR = 0.041). Conversely, a significant positive correlation was observed with acceleration capacity (ACmod: rho = 0.47, *p* = 0.030, p_FDR = 0.041).

In contrast, among patients with BC (*n* = 63), correlations between log-transformed extracellular DNA concentrations and the same HRV metrics were weak, inconsistent in direction, and non-significant after FDR correction.

To formally test whether this loss of correlation represents a statistically significant shift, we conducted a formal between-group comparison of the independent correlation coefficients using Fisher’s r-to-z transformation. The formal comparison revealed that the correlation coefficients for key vagal parameters differed significantly between healthy individuals and BC patients. Specifically, a significant difference was confirmed for DCmod (Spearman-adjusted z = −2.10, *p* = 0.036; unadjusted standard z = −2.16, *p* = 0.031) and RMSSD (Spearman-adjusted z = −2.03, *p* = 0.042; unadjusted standard z = −2.09, *p* = 0.036). The difference in correlation for ACmod was borderline significant under the conservative adjusted test (Spearman-adjusted z = 1.91, *p* = 0.056) and significant under the standard test (standard z = 1.97, *p* = 0.049). No significant differences between groups were observed for log HF (*p* = 0.123) or log LF (*p* = 0.881).

Taken together, these findings provide formal mathematical evidence of a loss of physiological coupling between autonomic regulation and circulating extracellular DNA homeostatic dynamics in patients with BC.

### 3.4. Survival Analysis

Patients with BC were stratified according to the optimal cutoff values derived from ROC analyses using the Youden index (extracellular DNA: 12.9 ng/mL and ncDNA: 3460 GE/mL). For mtDNA and DNase activity, ROC analyses demonstrated poor discriminatory performance (AUC = 0.535 and 0.522, respectively), and therefore no clinically meaningful ROC-derived cutoff values could be identified. No significant differences in overall survival (OS) were observed between the resulting subgroups (high ≥ cutoff vs. low < cutoff) using the log-rank test. Similar results were obtained when median values were used as cutoff points. These findings should be interpreted with caution given the relatively short follow-up period and the limited number of recorded events (death from any cause).

## 4. Discussion

### 4.1. Diagnostic Relevance of Circulating DNA Fractions

In this study, we demonstrated that circulating DNA markers are significantly altered in patients with urothelial carcinoma of the bladder. Both plasma extracellular DNA and ncDNA were elevated in patients compared to healthy controls. Notably, ncDNA showed the most consistent association with tumor stage, reaching its highest concentrations in patients with MIBC. In multivariable logistic regression analyses adjusted for demographic and lifestyle covariates, extracellular DNA remained independently associated with the presence of BC. This supports its potential diagnostic relevance, aligning with growing evidence for cell-free DNA (cfDNA) as a liquid biopsy biomarker in urothelial carcinoma [24,25].

The elevation of total extracellular DNA in BC patients is consistent with the concept that circulating DNA integrates multiple pathophysiological processes occurring in malignancy, including tumor cell turnover, stromal and immune-cell injury, necrosis, apoptosis, and inflammation-driven DNA release. However, total extracellular DNA is biologically heterogeneous and does not inherently distinguish tumor-derived DNA from host-derived DNA. This underlying heterogeneity may explain why extracellular DNA successfully differentiated patients from healthy controls and remained independently associated with cancer status, while its stage-related gradient was less robust than that observed for ncDNA.

The clearer association of ncDNA with MIBC suggests that the nuclear DNA fraction more closely reflects advanced tissue destruction, tumor burden, or accelerated cellular turnover. Importantly, circulating ncDNA may also contain tumor-derived DNA, which could make this fraction more directly related to malignant cell turnover and, in this sense, potentially more tumor-specific than total extracellular DNA. In contrast, mtDNA did not differ significantly between the groups in our cohort. Although circulating mtDNA has been proposed as an informative liquid biopsy component in various malignancies, recent data indicate that its diagnostic utility varies substantially depending on the tumor type and biological context [26].

### 4.2. Extracellular DNA Homeostasis and DNase Activity

DNase activity represents an important but often underappreciated component of extracellular DNA homeostasis. Reduced DNase activity may prolong the persistence of extracellular DNA and DNA–protein complexes in the circulation, thereby amplifying inflammatory, thrombotic, and immune-mediated effects. This is particularly relevant in the context of neutrophil extracellular traps (NETs), which are DNA-based structures increasingly implicated in cancer progression, immune evasion, metastatic dissemination, and treatment resistance. In experimental settings, degradation of NETs by DNase I has been explored as a strategy to attenuate pro-tumorigenic NET-associated effects [27,28].

In our cohort, DNase activity did not distinguish all patients with BC from healthy controls, but lower DNase activity in patients with MIBC compared with NMIBC may indicate impaired extracellular DNA clearance in more advanced disease. This finding should be interpreted cautiously because of the small MIBC subgroup, but it supports further investigation of DNase activity together with extracellular DNA fractions rather than as an isolated biomarker.

### 4.3. Autonomic–Inflammatory Coupling and HRV Dynamics

A novel exploratory aspect of this study was the analysis of associations between extracellular DNA and HRV parameters. In healthy individuals, higher extracellular DNA was associated with lower RMSSD, lower HF power, and lower DCmod, alongside higher ACmod. Because RMSSD and HF power predominantly reflect vagal modulation, these findings suggest a physiological relationship between circulating DNA and autonomic regulation, compatible with the concept that autonomic activity and inflammatory responses are interconnected via neuroimmune pathways.

Crucially, these associations were absent in patients with BC, and our formal between-group correlation analysis confirmed that the correlation coefficients for key vagal parameters (DCmod and RMSSD) were statistically significantly different between the healthy and patient cohorts. This formal statistical decoupling points to a disease-related loss of physiological oversight between autonomic regulation and extracellular DNA dynamics, potentially reflecting a disruption of the autonomic–inflammatory axis recently linked to neuroimmune regulation in cancer [18,19,20].

However, this interpretation remains hypothesis-generating and must be treated with caution due to several physiological and clinical factors. Heart rate variability is highly sensitive to a variety of confounding factors that were not fully controlled for in this exploratory study, including patient age, active medications (especially beta-blockers or other cardiovascular therapies), cardiac comorbidities, psychological stress, and individual respiratory patterns, which can directly affect vagal modulation. In a malignant state, systemic tumor-related stress, local inflammatory microenvironments, and metabolic alterations might disrupt or override the typical regulatory feedback loop of the cholinergic anti-inflammatory pathway. Future prospective trials with larger cohorts are necessary to adjust for these cardiovascular and clinical confounders through multivariate modeling, allowing for a more definitive validation of this autonomic–inflammatory decoupling in urothelial malignancy.

### 4.4. Methodological Considerations, and Limitations

Multivariable regression models strengthened the diagnostic validity of extracellular DNA by demonstrating that its association with BC persisted after adjustment for age, BMI, sex, smoking status, and alcohol consumption. This adjustment is particularly relevant since lifestyle factors like smoking can independently influence both BC risk and systemic inflammation. While the consistency across both raw and log-transformed extracellular DNA models supports the robustness of this finding, these models remain exploratory due to our modest sample size. The ncDNA model similarly suggested a positive association with BC status but was statistically constrained by missing data and quasi-complete separation. Thus, the ncDNA findings are best interpreted as a biologically plausible stage-related signal requiring validation in larger, more complete datasets.

Stratification by either median biomarker values or optimal cutoff values of extracellular DNA, ncDNA, mtDNA, or DNase activity did not reveal significant differences in overall survival. This negative finding prevents the overinterpretation of these circulating markers as established prognostic tools at this stage. Several factors may have obscured potential survival associations, including the small number of MIBC cases, low total event numbers, and the statistical limitations of median-based dichotomization, which can mask non-linear relationships. Furthermore, overall survival is heavily influenced by patient comorbidities and subsequent lines of therapy that may not be captured in baseline biomarker analysis. Additionally, a key analytical consideration involves the reliance on a single target marker for ncDNA (β-globin) and mtDNA (D-loop).

The modest sample size of our cohort (n = 84 total subjects, consisting of 21 healthy controls, 52 NMIBC, and 11 MIBC cases) represents a major limitation of this exploratory study. The relatively small number of subjects, particularly within the advanced MIBC subgroup, restricts the statistical power of subgroup comparisons and limits the generalizability of our findings. Consequently, the observed associations between circulating extracellular DNA fractions, DNase activity, and clinical stages must be interpreted with caution and treated as strictly hypothesis-generating.

Aside from the sample size constraints, additional limitations include the cross-sectional design, which precluded the assessment of temporal biomarker dynamics during treatment or recurrence. Furthermore, residual confounding from unmeasured comorbidities, renal function, or treatment heterogeneity cannot be entirely ruled out, and these circulating markers were not integrated with molecular tumor profiling or urinary biomarkers.

Although the loss of correlation between circulating cfDNA and HRV indices suggests an autonomic–inflammatory decoupling in patients with BC, we could not adjust for important physiological and clinical confounders of HRV—such as respiration rate, cardiovascular comorbidities, and the use of beta-blockers or other autonomic-modulating medications. Therefore, these findings must be considered preliminary. Finally, the use of single target markers for ncDNA and mtDNA introduces potential analytical limitations regarding fragment length or amplification biases.

### 4.5. Future Perspectives

To transition these exploratory biomarkers from pilot findings to clinically actionable tools, formal prospective validation in larger, well-powered, multi-center longitudinal cohorts is critically required. Such future studies will be essential to definitively establish the diagnostic, staging, and prognostic utility of circulating extracellular DNA in BC [29,30].

Future investigations should validate current findings using alternative reference markers (e.g., RNase P or GAPDH for nuclear DNA, and additional mitochondrial genes) to ensure broader analytical accuracy. Moreover, future studies evaluating circulating DNA in BC should explore potential correlations between fluorometrically measured total extracellular DNA and specific qPCR fractions, as well as assess the mitochondrial-to-nuclear DNA (mtDNA/ncDNA) ratio to gain deeper insights into relative cellular turnover and mitochondrial dynamics. Integrating circulating DNA findings into the broader context of precision oncology represents a promising avenue for translational research. Recent advancements in functional modeling, such as patient-derived BC organoids (PDOs), have provided robust platforms that faithfully recapitulate the histological and genomic heterogeneity of parental tumors [31,32]. Combining liquid biopsy approaches with patient-derived ex vivo models could bridge the gap between static biomarker detection and dynamic functional responses.

In addition, epigenetic regulators such as Enhancer of Zeste Homolog 2 (EZH2) play a pivotal role in cell-state remodeling, tumor plasticity, and disease progression in BC [33]. The dysregulation of chromatin modifiers frequently drives therapeutic resistance and aggressive phenotypes, which may be dynamically reflected in circulating nucleic acid profiles [34]. Future studies correlating circulating biomarker dynamics with EZH2-mediated expression patterns and organoid drug-response platforms will be essential to further advance personalized treatment strategies for BC patients.

## 5. Conclusions

Despite the limitations—most notably the modest sample size of our cohort and the exploratory nature of our clinical models—this pilot study provides a novel, integrated assessment of circulating extracellular DNA fractions, DNase activity, clinical stage, survival, and autonomic regulation in BC. Our findings support the value of extracellular DNA—and particularly ncDNA—as minimally invasive indicators of disease presence and biological severity, while also establishing DNase impairment and the loss of autonomic–inflammatory (HRV–extracellular DNA) coupling as compelling exploratory pathways of systemic dysregulation.

## Figures and Tables

**Table 1:** Characteristics of patients and tumors. Abbreviations: N: number of subjects; IQR: interquartile range; BMI: body mass index; NMIBC: non-muscle-invasive bladder cancer; MIBC: muscle-invasive bladder cancer; NA: not applicable.

	Patients	Controls	*p*-Value
*n* **(%)**	63 (100)	21 (100)	NA
Age (years)			
Median	68	67	
Range	40–85	40–79	0.672
IQR	11	21	
Sex			
Male, N, %	42 (66.7)	15 (71.4)	0.785
Female, N, %	21 (33.3)	6 (28.6)	
BMI (kg/m^2^)			
Median	27.5	25.8	
Range	18.6–39.5	19.3–34.4	0.154
IQR	5.5	3.7	
Smoking			
Current, N, %	19 (30.2)	3 (14.3)	
Former, N, %	20 (31.7)	3 (14.3)	0.030
None, N, %	24 (38.1)	15 (71.4)	
Stage			
Ta, N, %	23 (36.5)		
T1, N, %	29 (46.0)	NA	NA
NMIBC (Ta + T1), N, %	52 (82.5)		
MIBC (T2 + T3), N, %	11 (17.5)		

**Table 2:** Univariable and final multivariable logistic regression analysis of log-transformed extracellular DNA (extracellular DNA) and bladder cancer status.

Variable	Univariable OR (95% CI)	*p*-Value	Final Multivariable OR (95% CI)	*p*-Value
log extracellular DNA	3.88 (1.17–12.81)	0.026	5.99 (1.43–25.12)	0.015
Age (years)	1.02 (0.96–1.08)	0.534	–	–
BMI (kg/m^2^)	1.03 (0.92–1.16)	0.628	–	–
Sex	1.25 (0.42–3.69)	0.686	–	–
Smoking (past)	0.36 (0.10–1.36)	0.131	0.11 (0.02–0.54)	0.007
Smoking (current)	0.39 (0.10–1.47)	0.162	0.11 (0.02–0.59)	0.01
Daily alcohol consumption	2.87 (1.04–7.90)	0.042	3.92 (1.15–13.36)	0.029

Note: Univariable logistic regression analyses were performed for all candidate variables. A backward elimination procedure (removal criterion *p* > 0.10) was subsequently applied to obtain the final multivariable model. Age, BMI and sex were removed during the selection procedure. OR—odds ratio; CI—confidence interval; BMI—body mass index.

**Table 3:** Univariable and final multivariable logistic regression analysis of log-transformed nuclear circulating DNA (ncDNA) and bladder cancer status.

Variable	Univariable OR (95% CI)	*p*-Value	Final Multivariable OR (95% CI)	*p*-Value
log ncDNA	1.44 (1.01–2.06)	0.045	1.87 (1.01–3.47)	0.048
Age (years)	1.02 (0.96–1.08)	0.534	–	–
BMI (kg/m^2^)	1.03 (0.92–1.16)	0.628	–	–
Sex	1.25 (0.42–3.69)	0.686	–	–
Smoking (past)	0.36 (0.10–1.36)	0.131	0.19 (0.04–0.88)	0.034
Smoking (current)	0.39 (0.10–1.47)	0.162	0.17 (0.03–1.09)	0.061
Daily alcohol consumption	2.87 (1.04–7.90)	0.042	3.53 (0.98–12.76)	0.054

Note: This model included fewer observations due to missing data and exhibited quasi-complete separation, suggesting potential instability in the parameter estimates. Consequently, these results—particularly for categorical covariates—should be interpreted with caution. Univariable logistic regression analyses were performed for all candidate variables. A backward elimination procedure (removal criterion *p* > 0.10) was subsequently applied to obtain the final multivariable model. Age, BMI and sex were removed during the selection procedure. OR—odds ratio; CI—confidence interval; BMI—body mass index.

**Table 4:** Spearman correlations between circulating extracellular DNA and selected HRV parameters in healthy individuals and patients, with formal between-group correlation comparisons.

HRV Parameter	Healthy (n = 21) rho	Healthy *p*-Value	Healthy p_FDR	Patients (n = 63) rho	Patients *p*-Value	Patients p_FDR	Between-Group Difference (z-Score)	Between-Group Difference (*p*-Value) *
**DCmod (ms)**	−0.506	0.019	0.041	0.024	0.854	0.967	−2.101	0.036
**RMSSD (ms)**	−0.506	0.019	0.041	0.005	0.967	0.967	−2.032	0.042
**ACmod (ms)**	0.474	0.030	0.041	−0.014	0.914	0.967	1.913	0.056
**log HF**	−0.468	0.033	0.041	−0.081	0.530	0.967	−1.541	0.123
**log LF**	−0.216	0.348	0.348	−0.255	0.045	0.225	0.149	0.881

Note: * *p*-values for between-group differences were derived from the Spearman-adjusted Fisher’s r-to-z test statistic incorporating the 1.06 non-parametric variance correction factor. Abbreviations: DCmod; deceleration capacity; Acmod, acceleration capacity; RMSSD, root mean square of successive differences; log LF, log-transformed low-frequency power; log HF, log-transformed high-frequency power.

## Data Availability

The original contributions presented in this study are included in the article. Further inquiries can be directed to the corresponding authors.
